# *Mycoplasma bovis* inhibits autophagy in bovine mammary epithelial cells *via* a PTEN/PI3K-Akt-mTOR-dependent pathway

**DOI:** 10.3389/fmicb.2022.935547

**Published:** 2022-07-26

**Authors:** Maolin Xu, Yang Liu, Tuerdi Mayinuer, Yushan Lin, Yue Wang, Jian Gao, Dong Wang, John P. Kastelic, Bo Han

**Affiliations:** ^1^Department of Clinical Veterinary Medicine, College of Veterinary Medicine, China Agricultural University, Beijing, China; ^2^College of Life Science, Ningxia University, Yinchuan, China; ^3^Department of Production Animal Health, Faculty of Veterinary Medicine, University of Calgary, Calgary, AB, Canada

**Keywords:** *Mycoplasma bovis*, bovine mammary epithelial cells, autophagy, PI3K-Akt-mTOR pathway, PTEN

## Abstract

Although autophagy can eliminate some intracellular pathogens, others, e.g., *Staphylococcus aureus*, Salmonella, *Mycoplasma bovis*, can evade it. The phosphoinositide 3-kinase (PI3K)/protein kinase B (Akt)/mammalian target of rapamycin (mTOR) pathway, a key regulator of autophagy, is involved in initiation and promotion of a range of pathological diseases. As the effects of *M. bovis* on the autophagic pathway are not well documented, our objective was to elucidate the effects of *M. bovis* infection on the PI3K-Akt-mTOR cellular autophagic pathway in bovine mammary epithelial cells (bMECs). Ultrastructure of bMECs infected with *M. bovis* was assessed with transmission electron microscopy, co-localization of LC3 puncta with *M. bovis* was confirmed by laser confocal microscopy, and autophagy-related indicators were quantified with Western blotting and RT-PCR. In *M. bovis*-infected bMECs, intracellular *M. bovis* was encapsulated by membrane-like structures, the expression level of LC3-II and Beclin1 protein decreased at the middle stage of infection, degradation of SQSTM1/P62 was blocked, autophagy of bMECs was inhibited, and PI3K-Akt-mTOR protein was activated by phosphorylation. Furthermore, the tumor suppressor PTEN can inhibit the PI3K-Akt signaling pathway through dephosphorylation of phosphatidylinositol 3,4,5-trisphosphate and may be important for cellular resistance to infection. In the present study, the number of intracellular *M. bovis* was inversely related to the change in the level of autophagy markers (e.g., LC3-II, SQSTM1/P62) within host cells induced by the low knockdown of Akt or PTEN. We concluded that *M. bovis*-infected bMECs alleviated cellular autophagy through a PI3K-Akt-mTOR pathway, and that PTEN acted as a protective gene regulating autophagy, a key step in controlling infection.

## Introduction

*Mycoplasma bovis* is a devastating pathogen in dairy cows worldwide, causing pneumonia, arthritis, mastitis, and keratoconjunctivitis (Maunsell and Chase, 2019). Its control is complicated by increasing antimicrobial resistance and a lack of effective vaccines (Gautier-Bouchardon et al., 2014; Perez-Casal, 2020). Therefore, better understanding of *M. bovis* pathogenesis and immune evasion are needed for evidence-based control.

Autophagy is a lysosome-dependent and ubiquitous self-digestion process in eukaryotic cells (Kim et al., 2018). In addition, intracellular pathogens can be targets of selective autophagy (xenophagy) that recognizes, captures, and transports heterologous components to lysosomes for degradation to control infection (Mostowy, 2013). Microtubule-associated protein light chain 3 (Atg8/LC3) is the most widely monitored autophagy-related protein, the protein diffusely distributed in the cytoplasm, is linked to target substrates (Mizushima and Yoshimori, 2007). The SQSTM1/P62 protein serves as a link between LC3 and ubiquitinated substrates. SQSTM1 and SQSTM1-bound polyubiquitinated proteins become incorporated into the completed autophagosome and are degraded in autolysosomes, thus serving as a readout of autophagic degradation (Bjørkøy et al., 2005). BECN1/Atg6 and PIK3C3/VPS34 are essential partners in the autophagy interactome that signals the onset of autophagy, and many researchers use BECN1 as a way to monitor autophagy (Yang and Klionsky, 2010). However, some pathogens can evade degradation by autophagy or even inhibit autophagy (Choy and Roy, 2013; Huang and Brumell, 2014). For example, *M. bovis* can invade various host cells, including epithelial or immune cells (Josi et al., 2018; Liu et al., 2020). In addition, *Mycoplasma hyopneumoniae* infection can induce incomplete autophagy in host cells, which enhances its ability to multiply in host cells (Wang et al., 2021b). Furthermore, we reported *M. bovis* promoted replication by blocking its delivery to autophagosomes and lysosomes (Liu et al., 2021).

Several cell signaling pathways are linked to regulation of autophagy, including the phosphoinositide 3-kinase/protein kinase B (Akt)/mammalian target of rapamycin (PI3K-Akt-mTOR) signaling pathway. Furthermore, the first class of PI3Ks (PI3K-I) activates Akt and mTOR activity to inhibit cellular autophagy by triggering phosphorylation of Akt and mTOR (Farrell et al., 2013). Inhibition of the PI3K-Akt-mTOR signaling pathway can affect post-infection inflammatory, apoptotic, and autophagic responses, protecting host cells from various pathogens, including *Mycobacterium tuberculosis* (Lachmandas et al., 2016), *Legionella pneumophila* (Abshire et al., 2016), and *Listeria monocytogenes* (Gessain et al., 2015). In addition, *M*. *bovis* infection of dairy mammary tissue up-regulated the mRNA expression level of PI3K-Akt signaling pathway (Özdemir and Altun, 2020).

The phosphatase and tensin homolog deleted on chromosome ten (PTEN), a lipid phosphatase, inhibits the PI3K-Akt-mTOR signaling pathway, mainly through dephosphorylation of phosphatidylinositol 3,4,5-trisphosphate; furthermore, expression of exogenous PTEN in PTEN mutant cells restores the normal pattern of PKB/Akt phosphorylation (Dempsey et al., 2021; He et al., 2021). A deficiency of PTEN hypersensitizes multiple cell types to *Mycoplasma* and *Mycobacterium bovis* infections and the lipid phosphatase activity of PTEN is required to attenuate infection (Huang et al., 2012). Thus, influencing cellular autophagy by modulating key autophagic signaling pathways is an important novel clearance strategy against intracellular pathogen invasion. However, effects of *Mycoplasma* infection on autophagy signaling pathways are not well understood.

The objective was to determine the role of PTEN/PI3K-Akt-mTOR signaling pathway in *M. bovis* infection, using an *in vitro* model with bovine mammary epithelial cells (bMECs). This is apparently the first report showing that *M. bovis* regulates cellular autophagy through the PTEN/PI3K-Akt-mTOR pathway.

## Materials and methods

### Statement of ethics

This study was conducted in accordance with ethical guidelines and standard biosecurity and institutional safety procedures of China Agricultural University (CAU; Beijing, China). Prior to the start of the study, ethical approval was granted by the Departmental Committee of the College of Veterinary Medicine, CAU.

### Antibodies and reagents

Enhanced Cell Counting Kit-8 (CCK-8), Ad-GFP-LC3B, DiI (1,1′-dioctadecyl-3,3,3′,3′-tetramethylindocarbocyanine perchlorate), Bicinchoninic acid (BCA) protein assay kit, and radioimmunoprecipitation assay (RIPA) lysis buffer (Beyotime Biotechnology) were all purchased from Beyotime (Shanghai, China). The pleuropneumonia-like organism (PPLO) broth was from BD Biosciences (San Jose, CA, United States), whereas Fetal Bovine Serum (FBS), Dulbecco’s modification of Eagle’s medium (DMEM) with high glucose and Opti-MEM were from Gibco (Grand Island, NY, United States). Horse serum was purchased from Hyclone (Logan, UT, United States). Penicillin G, amphotericin and bovine serum albumin (BSA) were from Coolaber (Beijing, China). Dimethyl sulfoxide (DMSO) was acquired from Sigma-Aldrich Chemical (Sigma, St. Louis, MO, United States). The pan-ErbB inhibitor CI-1033 was purchased from MedChemExpress (Monmouth Junction, NJ, United States). TransScriptFirst-Strand complementary DNA (cDNA) Synthesis SuperMix and SYBRGreen PCR Core Reagents were purchased from TransGenBiotech (Beijing, China). Trizol Reagent was bought from Invitrogen (Carlsbad, CA, United States). Small interfering RNA (siRNA) against the Akt, PTEN, negative-control siRNA, and siRNA-mate transfection reagent were all purchased from GenePharma (Shanghai, China). Polyvinylidene difluoride membrane was from Millipore (Bedford, MA, United States). Glutaraldehyde, 2.5% (EM Grade), coverslips, and 4′,6-Diamidine-2′-phenylindole dihydrochloride (DAPI) were all purchased from Solarbio (Beijing, China). An enhanced chemiluminescence (ECL) kit was obtained from Thermo Fisher Scientific Pierce (Rockford, IL, United States). Anti-PTEN antibody (Catalogue number: 22034-1-AP), anti-mTOR antibody (Catalogue number: 28273-1-AP), anti-Beclin1 antibody (11306-1-AP), anti- Sequestosome 1 (SQSTM1/p62) antibody (Catalogue number: 18420-1-AP), anti-β-actin antibody (Catalogue number: 66009-1-Ig), anti-glyceraldehyde 3-phosphate dehydrogenase (GAPDH) antibody (Catalogue number: 60004-1-Ig), anti-mouse Ig G–horseradish peroxidase (HRP; Catalogue number: SA00001-1), and Goat anti-rabbit IgG (Catalogue number: SA00001-2) were all purchased from Proteintech (Chicago, IL, United States). Anti-LC3B antibody (#3868), anti-Akt antibody (#9272), Anti-p-PI3 Kinase p85 (Tyr458)/p55 (Tyr199; #17366), Anti-p-Akt (Ser473; #4060), Anti-p-mTOR (Ser2448; #5536) were from Cell Signaling Technology (Danvers, MA, United States).

### *Mycoplasma bovis* strain and cell culture

*M. bovis* strain PG45 (ATCC 25523) was purchased from the ATCC. For infection experiments, *M. bovis* was cultured in PPLO broth with 20% horse serum and penicillin (100 IU/l) in 5% CO_2_ at 37°C for 72 h. The PPLO broth was prepared by dissolving 10.5 g of Difco PPLO medium (BD Biosciences) and 1 g of yeast extract (BD Biosciences) in 400 ml ultrapure water, then autoclaving it at 121°C for 30 min. *M. bovis* was collected by centrifugation (6000 × g for 15 min) and then washing with phosphate-buffered saline (PBS). The number of colony forming units was determined by performing 10-fold serial dilutions in PBS and subsequently spotting on PPLOA plates (Bürki et al., 2015), prepared by supplementing PPLO broth with 20% horse serum and 0.75% agar. Bacteria were suspended in PBS to a cell density of 10^8^ colony-forming units per milliliter (CFU/mL), and the suspension was stored at −70°C until use (Gondaira et al., 2021). A line of bovine mammary epithelial cells (bMECs; MAC-T; Shanghai Jingma Biological Technology Co., Ltd., Shanghai, China) was cultured in cell culture plates (Corning Inc., Corning, NY, United States) in DMEM supplemented with 10% FBS at 37°C with 5% CO_2_. In all experiments, bMECs were allowed to grow and adhere for 24 h in culture medium prior to being infected with *M. bovis.* The bMECs were seeded at a concentration of 1 × 10^5^ cells/mL in 6-well plates (2 ml per well) 24 h prior to experiments.

### Cell infection and gentamicin protection assay

When bMECs cultured in a 6-well plate reached 60–70% confluence, the bMECs were inoculated (time = 0 h) with *M. bovis* PG45 strain at a multiplicity of infection (MOI) of 1:30. After infection at 37°C for 1 h, the inoculum was removed and cells were washed twice with autoclaved PBS, 2 ml/well, to remove non-adherent *M. bovis*. Thereafter, all extracellular *M. bovis* was killed by adding 2 ml/well DMEM with 400 μg/ml gentamicin for 2 h at 37°C (time = 2 h). Cells were again washed as described above. Finally, fresh DMEM with 10% fetal bovine serum (FBS) and 10 μg/ml gentamicin was added to the infected cells, 2 ml/well (time = 1 h).

### Enumerating intracellular *Mycoplasma bovis*

At designated time points, cells were washed 3 times with PBS after treatment, as described above, and CFU enumerated as described (Bürki et al., 2015). Cells were detached from plates with a 23-gauge needle and syringe, and bacterial concentrations confirmed by plating 10-fold serial dilutions (Bürki et al., 2015). To enumerate CFU, *M. bovis* in each cell well were counted, with six repeats on plates.

### Transfection

Transfection with GFP-LC3B has been widely used to monitor autophagy or co-localization with cargo (Klionsky et al., 2021). Ad-GFP-LC3B, an adenovirus expressing GFP-LC3B fusion protein (Klionsky et al., 2021), was used, in accordance with manufacturer’s instructions, to transfect bMECs. Briefly, bMECs were seeded on 6-well plates with coverslips, followed by incubation in 5% CO_2_ at 37°C for 12 h. When bMECs density was 40–50% confluence, cells were infected with Ad-GFP-LC3B at MOI of 1:10 in DMEM containing 10% FBS. Then, cells were incubated in 5% CO_2_ at 37°C for 24 h.

To knock down Akt and PTEN, specific small interfering RNA (siRNA) duplexes targeting the bovine Akt and PTEN gene and 2 siRNA negative controls were purchased from GenePharma (Shanghai, China). First, 2.5 nmol Akt and PTEN siRNA and control siRNA were dissolved in 125 μl of DEPC water and stored at −70°C. Then, Akt and PTEN siRNA and control siRNA stock solution were diluted in Opti-MEM medium and diluted siRNA was added to siRNA-mate reagent and incubated for 10 min in Opti-MEM (2 μl of Akt and PTEN siRNA and control siRNA, 200 μl of Opti-MEM medium, and 4 μl of siRNA-mate reagent). Cells were treated with an siRNA directed against Akt, PTEN and the scrambled control siRNA (final concentrations, 25 nM) using siRNA-mate reagent in Opti-MEM without serum, according to manufacturer’s instructions. After 6 h, transfection medium was removed and 2 ml of DMEM supplemented with 10% FBS at 37°C with 5% CO_2_ was added to recover cell growth for 48 h. Subsequently, cells were infected with *M. bovis*.

### Induction of autophagy

Epidermal growth factor receptor (EGFR) is located on the cell surface and induces PI3K activation. To reduce PI3K signaling in bMECs without completely inhibiting intracellular PI3K function, we selected the pan-ErbB inhibitor Canertinib (CI-1033), a potent EGFR inhibitor, to treat cells prior to infection. The bMECs were inoculated into 6-well plates for 24 h, CI-1033 (1 μM) was added to the culture medium and co-cultured for 1 h. Moreover, effects of CI-1033 on *M. bovis* viability were determined. Specifically, 1 × 10^6^ CFU/ml *M. bovis* was incubated in PPLO medium with 20% horse serum, with or without CI-1033. The CFU of *M. bovis* was determined as described above, after incubation in 5% CO_2_ at 37°C for 24 h. Effects of CI-1033 on viability of bMECs were evaluated with CCK-8 assays (Geng et al., 2020). Briefly, bMECs were seeded in 96-well plates (1 × 10^4^ cells/well) in 100 μl DMEM with 10% FBS. After incubation in 5% CO_2_ at 37°C for 24 h, cells reached 60–70% confluence. Cells were treated with CI-1033 (1 μM in DMEM with 10% FBS) or nothing (Control), followed by 24 h incubation in 5% CO_2_ at 37°C. Then, fresh DMEM containing 10% CCK-8 was placed in the well, cells were incubated in 5% CO_2_ at 37°C for 2 h and optical density was determined with a microplate reader (Bio-Rad, Hercules, CA, United States) at 450 nm. The density of treated cells relative to control cells was calculated.

### Western blotting

At designated time points, cells were washed 3 times with PBS and total protein extracted with RIPA lysis buffer on ice. The liquid was centrifuged at 12,000 × g for 20 min. Protein concentrations were determined with a BCA protein assay kit, according to manufacturer’s instructions. For each sample, equal amounts of protein were separated by electrophoresis on 8 and 12% sodium dodecyl sulfate-polyacrylamide gels (SDS-PAGE). Subsequently, proteins were transferred onto a PolyVinyliDene Fluoride (PVDF) using a semi-dry blotting system. Blots were first blocked with 5% skim milk in 0.1% Tris buffered saline-Tween-20 (TBST), pH 7.4 at room temperature for 2 h, followed by 3 washes in TBST for 10 min each. After blocking, membranes were incubated with specific primary antibodies overnight at 4°C. After 3 washes in TBST for 10 min each, membranes were probed with HRP-conjugated secondary antibody for 1 h at room temperature. Signals were detected using an ECL-Plus Western blot detection system and band density analyzed with ImageJ (National Institutes of Health, Bethesda, MD, United States).

### RNA extraction, cDNA synthesis and real-time PCR

At the various time points indicated, bMECs were harvested with 1 ml TransZol Up lysis solution and total RNA was extracted with a total RNA extraction kit (TransGen Biotech), according to manufacturer’s instructions. The cDNA was synthesized using TransScript^®^ II All-in-One First-Strand cDNA Synthesis SuperMix for qPCR (One-Step gDNA Removal; TransGen Biotech). To verify that the extracted RNA was free of DNA contamination, a control group without reverse transcriptase was set up while reverse transcribing the RNA samples into cDNA, which was detected by PCR amplification and 1% agarose gel electrophoresis to ensure the absence of DNA contamination. Both RNA and cDNA were quantified with a NanoDrop One spectrophotometer (Thermo Fisher Scientific, Waltham, MA, United States). For autophagy-associated genes, mRNA expression levels were verified with real time PCR, as follows: pre-denaturation at 94°C for 2 min, followed by 40 cycles of denaturation at 95°C for 5 s, and annealing at 60°C for 60 s using the Applied Biosystems StepOnePlus Real Time PCR system (Thermo Fisher Scientific). For melt curve analysis, PCR products were heated from 55 to 95°C, with the fluorescence signal assessed every 0.5°C to verify primer specificity. Cycle threshold (Ct) values were determined with StepOne TM Software version 2.3 (Thermo Fisher Scientific). In this study, ΔCt = Ct target gene−Ct endogenous control (arithmetic mean of the reference gene), whereas ΔΔCt = ΔCt sample − Ct control (uninfected cells). To visualize impacts of *M. bovis* on responses of target genes in bMECs, relative mRNA expression data were presented as 2 − ΔΔCt. Real-time quantitative PCR was used to amplify 100 ng of cDNA using the following pairs of primers: cattle Akt upstream primer 5′-ggcacatcaagatcaccgac −3′ and down-stream primer 5′-tcctggttgtagaagggcag-3′ (NCBI Reference Sequence: NM_173986.2); cattle SQSTM1/P62 upstream primer 5′-tctgccctgactacgaccta-3′ and down-stream primer 5′-cccaaagtgcccatgtttca-3′ (NCBI Reference Sequence: NM_176641.1); cattle LC3B upstream primer 5′-gtccgacttatccgagagca-3′ and down-stream primer 5′-tggacacactcaccatgcta-3′ (NCBI Reference Sequence: NM_001001169.1); cattle Beclin1 upstream primer 5′-actggacacgagcttcaaga-3′ and down-stream primer 5′-agatgcctccccaatcagag-3′ (NCBI Reference Sequence: NM_001033627.2); cattle GAPDH upstream primer 5′-aaggccatcaccatcttcca-3′ and down-stream primer 5′-tcacgcccatcacaaacatg-3′ (NCBI Reference Sequence: NM_001034034.2). For these studies, GAPDH was the reference gene.

### Transmission electron microscopy

The bMECs were removed with a cell scraper and centrifuged at 1000 × g for 5 min. Cells were washed twice with PBS, fixed with 2.5% glutaraldehyde for ≥2 h, then fixed in 1% osmium tetroxide for 2 h at 4°C. After dehydration in a graded ethanol series, samples were embedded in epoxy resin-acetone mixtures for 2 h, followed by immersion in a pure resin solution overnight at 37°C. After polymerization, ultrathin sections (50 ~ 70 nm) were cut, stained with saturated uranyl acetate in 50% ethanol and lead citrate, and examined with a transmission electron microscope (JEM-1400, JEOL, Tokyo, Japan).

### Confocal laser microscopy inspection

To assess co-localization of autophagy-associated genes with intracellular *M. bovis*, LC3B and *M. bovis* were detected by confocal laser microscopy (Liu et al., 2021). *M. bovis* was collected by centrifugation (6,000 × g, 15 min) washed with PBS, pre-stained with DiI cell membrane red fluorescent probe (10 μM) for 20 min, protected from light, centrifuged again at 6000 × g for 15 min, washed 3 times with PBS, resuspended in DMEM, and then used to infect bMECs that had been pre-seeded in 6-well plates containing coverslips. Cells on coverslips were fixed with 4% paraformaldehyde for 15 min at room temperature, then washed 3 times with PBS. Finally, coverslips were stained with fluorescence mounting medium containing DAPI and mounted on glass slides. Images were captured with a Nikon A1 LFOV confocal microscope at laser wavelengths of 405, 561 and 488 nm. Pixel co-localization analysis of specific regions within cells was performed using Pearson’s correlation coefficient using Image J software with JaCoP plug-in. Representative cells were selected and photographed. There were 20 cells per sample and at least 60 cells per group for statistical analysis.

### Statistical analyses

**Figure 1:**
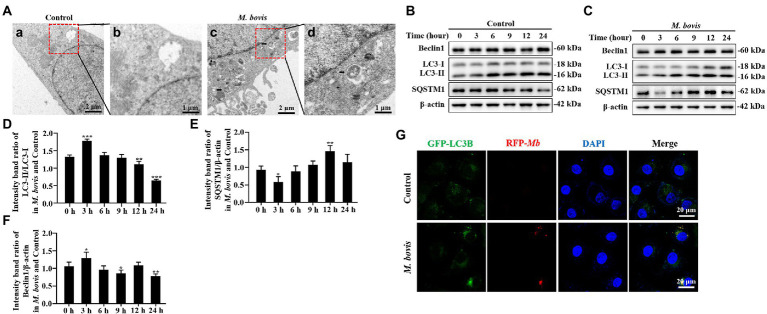
*M. bovis* infection induced autophagy in bMECs. **(A)** Ultra-microstructure observations of intracellular *M. bovis*, bMECs control (a) or *M. bovis* strain PG45 infected at an MOI of 30 for 3 h (c); the black arrow is an autophagosome-like membrane vesicle. Control and *M. bovis*-infected cells were fixed and processed for electron microscopy. Scale bars, 2  μm (a,c) and 1  μm (b,d). **(B,C)** bMECs were control or infected with *M. bovis* (MOI = 30) for 0, 3, 6, 9, 12, and 24  h. At the end of the infection, expression levels of LC3, SQSTM1, Beclin1, ATG5, and β-actin (loading control) were analyzed by Western blotting with specific antibodies. **(D–G)** Relative quantification of LC3-II, SQSTM1, Beclin1, ATG5 protein levels compared to LC3-I or β-actin protein levels was determined by densitometry, and the ratio of *M. bovis* infected group to control group protein levels was calculated. **(H)** Ad-GFP-LC3B transfected bMECs for 24  h and then cells were infected with *M. bovis* (stained red with DiI, MOI = 30). Cells were fixed and nuclei counterstained with DAPI prior to confocal laser microscopy. Scale bars: 20  μm. Data represent mean ± SD of 3 independent experiments. ^*^p < 0.05; ^**^p < 0.01; ^***^p < 0.001.

**Figure 2:**
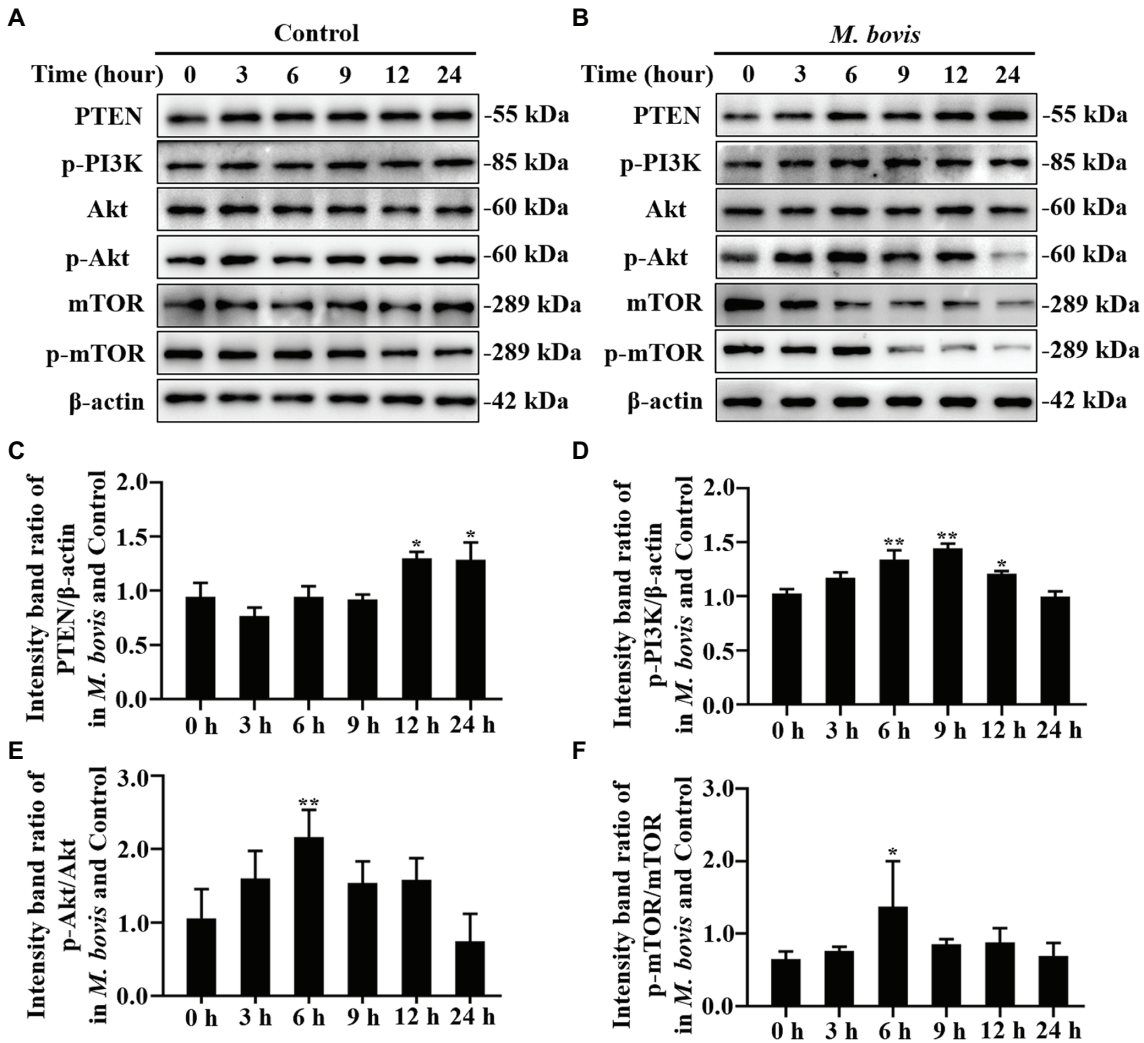
The role of PTEN/PI3K-Akt-mTOR pathway in *M. bovis* affecting autophagy in bMECs. **(A,B)** bMECs were control or infected with *M. bovis* (MOI = 30). Cells were harvested at 0, 3, 6, 9, 12, and 24  h and then Western blotted with anti-PTEN, anti-p-PI3K, anti-p-mTOR, anti-mTOR, anti-p-Akt, anti-Akt, and anti-β-actin (loading control) antibodies. **(C)** PTEN protein levels relative to β-actin were determined by densitometry. **(D)** p-PI3K protein levels relative to β-actin were determined by densitometry. **(E)** p-mTOR levels relative to mTOR were determined by densitometry. **(F)** p-Akt levels relative to Akt were determined by densitometry. The ratio of *M. bovis* infected group to control group protein levels was calculated. Data represent the mean ± SD of 3 independent experiments. ^*^p < 0.05; ^**^p < 0.01.

**Figure 3:**
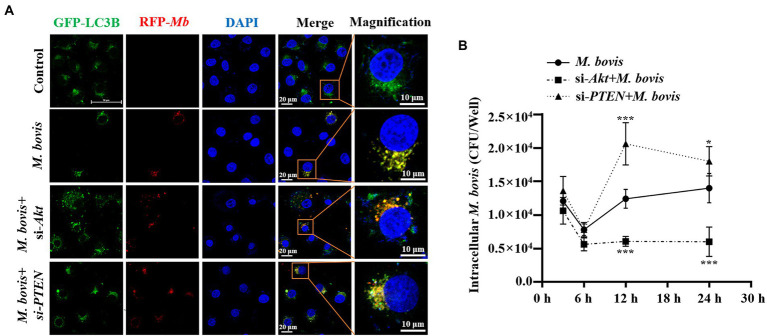
PTEN promoted autophagy of bMECs by inhibiting PI3K-Akt–mTOR pathway to suppress replication of *M. bovis*. **(A)** bMECs were transfected with siRNA-Akt (25  nM) and siRNA-PTEN (25  nM) for 48  h and then infected with *M. bovis* (MOI = 30). Co-localization of LC3B (green) with *M. bovis* (red) was detected by confocal microscopy at 6  h post-infection. Cell nuclei were counterstained with DAPI. Scale bar: 10 and 20  μm. **(B)** Comparison of intracellular replication of *M. bovis* in bMECs with downregulated Akt or PTEN status. bMECs were transfected with siRNA-Akt (25  nM) and siRNA-PTEN (25  nM) for 48  h and then infected with *M. bovis* (MOI =30); the intracellular *M. bovis* load was measured at 3, 6, 12, and 24  h postinfection. Data represent mean ± SD of 3 independent experiments. ^*^p < 0.05; ^***^p < 0.001.

**Figure 4:**
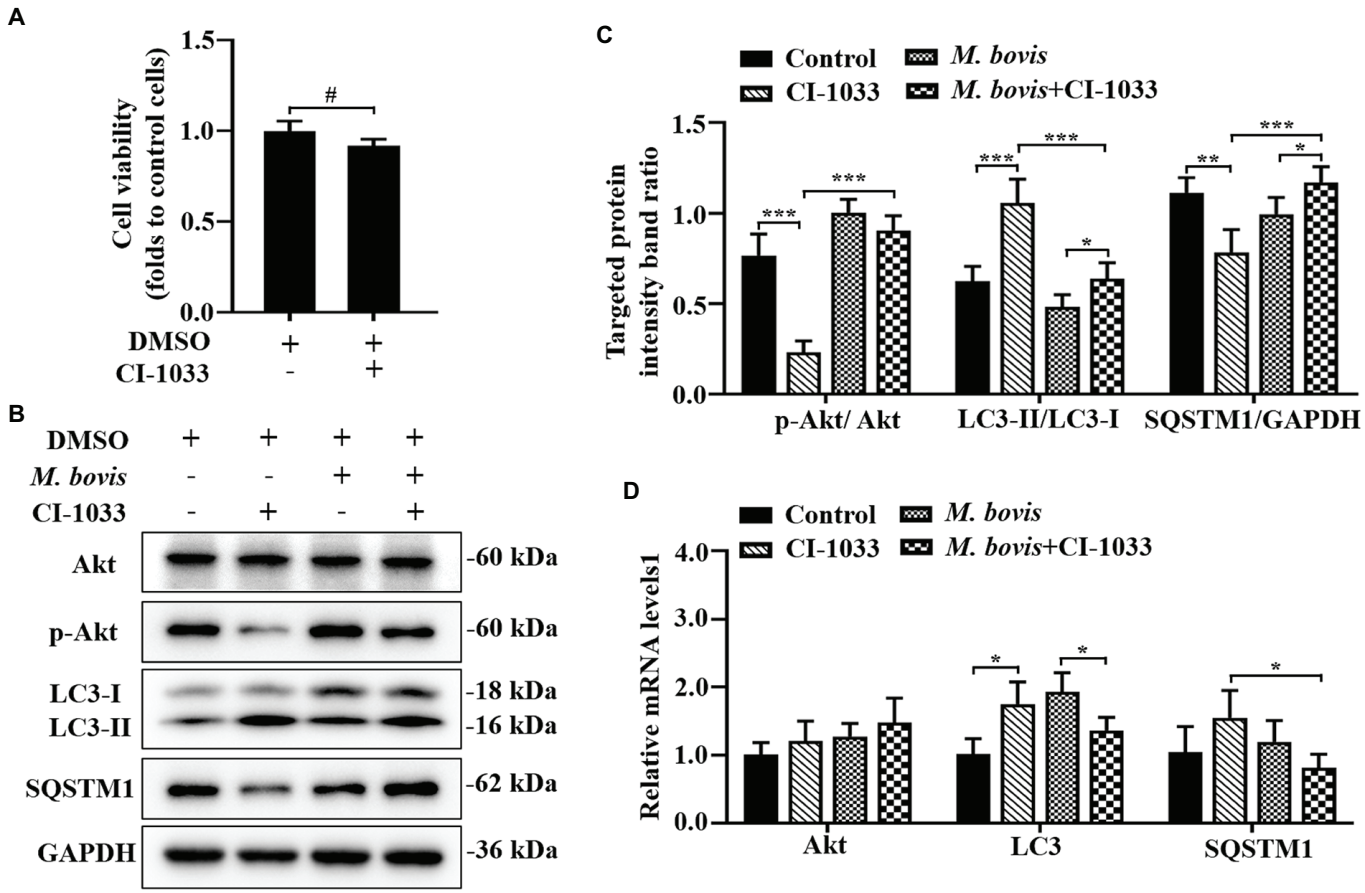
Phosphorylation of Akt was positively related to inhibition of autophagy by *M. bovis*. **(A)** Effects of CI-1033 on viability of bMECs in DMEM medium containing 10% FBS based on CCK-8 assays. **(B)** Cells were pre-treated with CI-1033 (1  μm) for 1  h and then control with DMSO, and cells infected with *M. bovis* (MOI = 30) were further cultured for 6  h. Thereafter, cell samples were analyzed by Western blotting with anti-Akt, anti-p-Akt, anti-LC3, anti-SQSTM1, and anti-GAPDH (loading control) antibodies. **(C)** Relative quantification of p-Akt protein levels compared to Akt protein, LC3-II protein levels compared to LC3-I protein, and SQSTM1 protein levels compared to GAPDH protein was determined by densitometry in *M. bovis*-infected bMECs in the absence or presence of CI-1033. **(D)** Transcriptional levels of PI3K-Akt–mTOR signaling pathway and autophagy-related genes including Akt, LC3-II and SQSTM1, were detected by real-time PCR in bMECs. The data represent the mean ± SD of 3 independent experiments. ^*^p < 0.05; ^**^p < 0.01; ^***^p < 0.001.

**Figure 5:**
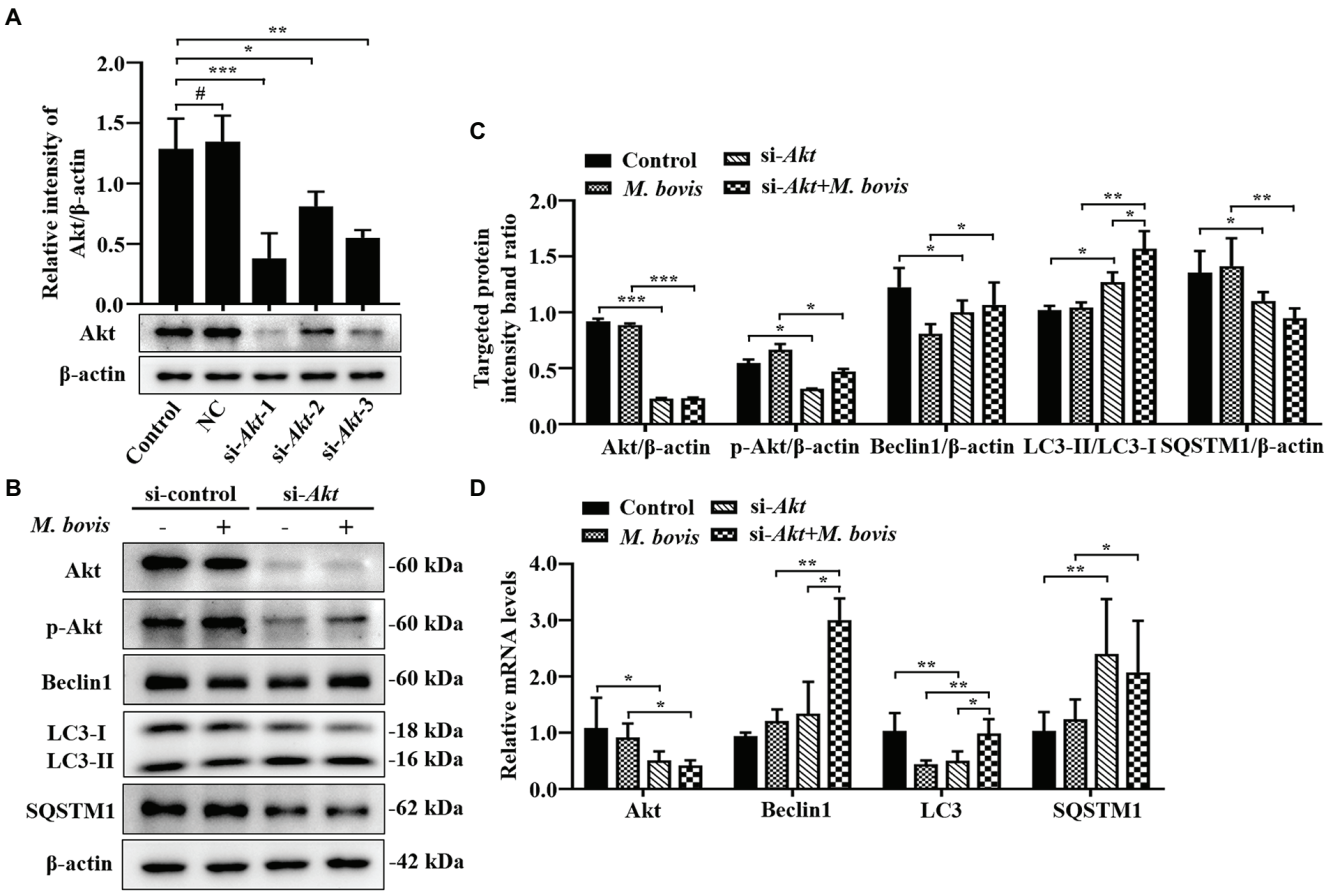
Inhibition of Akt with specific siRNAs targeting Akt reversed inhibition of autophagy in bMECs by *M. bovis*. **(A)** bMECs were transfected with siRNA-control (NC; 25  nM) and siRNA-Akt-1/2/3 (25  nM) for 48  h. Akt protein expression were analyzed by Western blotting. **(B)** bMECs were transfected with siRNA-Akt-1 targeting Akt for 48  h; then, cells were infected with *M. bovis* (MOI = 30) for 6  h. Thereafter, cell samples were analyzed by Western blotting with anti-Akt, anti-p-Akt, anti-LC3, anti-SQSTM1, and anti-β-actin (loading control) antibodies. **(C)** Relative quantification of target protein levels compared to β-actin protein was determined by densitometry in transfected siRNA-Akt-1 cells. **(D)** Transcriptional levels of PI3K-Akt–mTOR signaling pathway and autophagy-related genes including Akt, LC3-II and SQSTM1, were detected by real-time PCR in bMECs. Data represent mean ± SD of 3 independent experiments. ^#^*p* > 0.05; ^*^p < 0.05; ^**^p < 0.01; ^***^p < 0.001.

**Figure 6:**
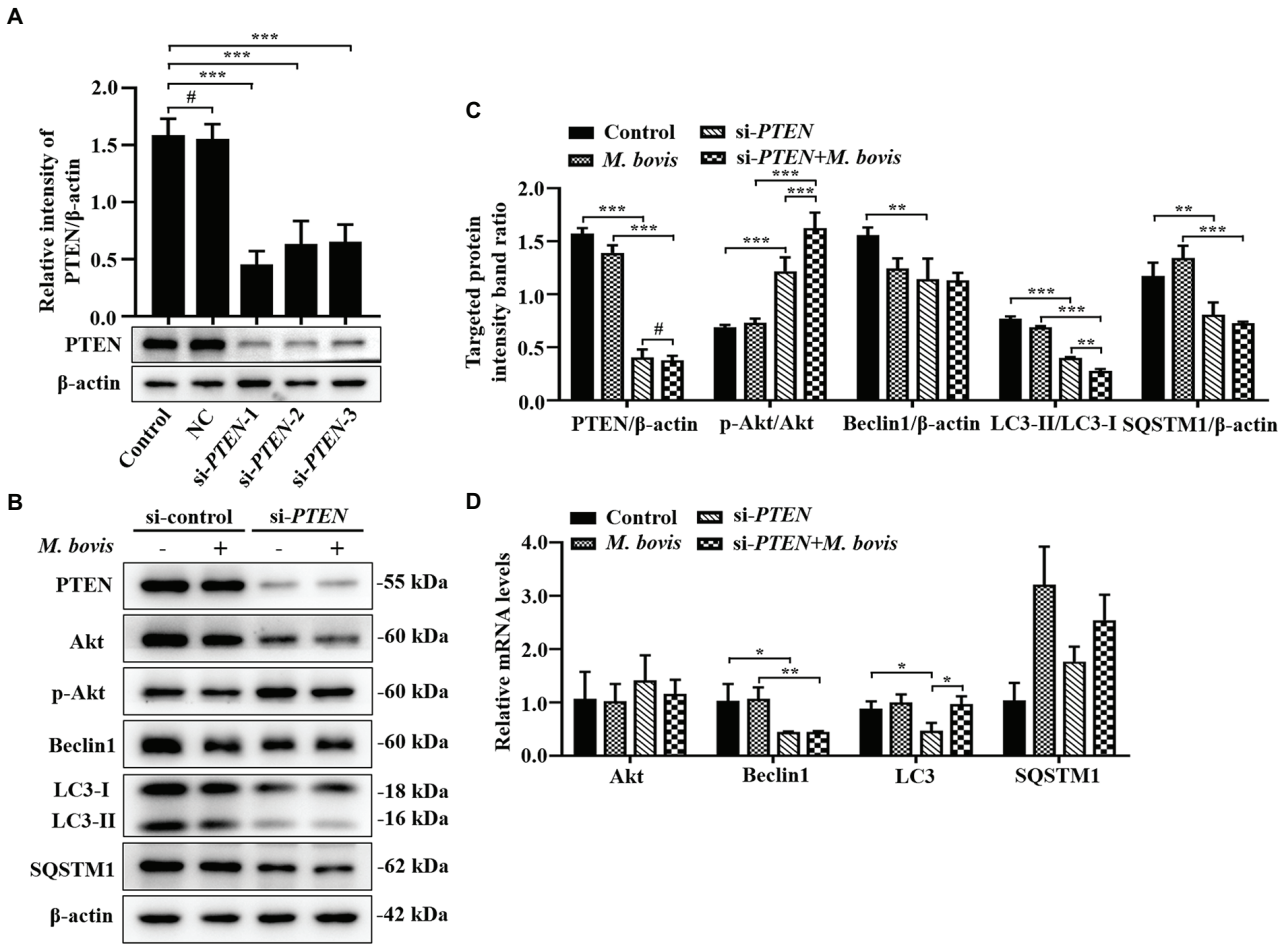
Absence of PTEN expression exacerbated inhibition of autophagy of bMECs by *M. bovis*. **(A)** bMECs were transfected with siRNA-control (NC; 25  nM) and siRNA-PTEN-1/2/3 (25  nM) for 48  h. PTEN protein expression was analyzed by Western blotting. **(B)** bMECs were transfected with siRNA-PTEN-1 targeting PTEN for 48  h; then, cells were infected with *M. bovis* (MOI = 30) for 6  h. Thereafter, cell samples were analyzed by Western blotting with anti-PTEN, anti-Akt, anti-p-Akt, anti-Beclin1, anti-LC3, and anti-β-actin (loading control) antibodies. **(C)** Relative quantification of target protein levels compared to β-actin protein was determined by densitometry in transfected siRNA-PTEN-1 cells. **(D)** Transcriptional levels of PI3K-Akt–mTOR signaling pathway and autophagy-related genes including Akt, Beclin1 and LC3-II, were detected by real-time PCR in bMECs. Data represent the mean ± SD of 3 independent experiments. ^#^*p* > 0.05; ^*^p < 0.05; ^**^p < 0.01; ^***^p < 0.001.

All assays were repeated 3 times independently, unless otherwise stated. To calculate the *p* values, a one-way analysis of variance (ANOVA) with Dunnett’s *post-hoc test* was used for Figures 1D–F, 2C–F, 3B, whereas a two-way ANOVA with Bonferroni posttest was used for Figures 4C,D, 5C,D, 6C,D. All statistical analyses were done with SPSS 26.0 software (IBM Corp., Armonk, NY, United States) and histograms produced with GraphPad Prism 8.0 (GraphPad Software, Inc., San Diego, CA, United States). Data were expressed as mean ± standard deviation (SD) and *p* < 0.05 was considered significant. Significant differences were designated as follows: ^#^*p* > 0.05; ^*^p < 0.05; ^**^p < 0.01; and ^***^p < 0.001.

## Results

### *Mycoplasma bovis* infection increases the levels of autophagic markers in bMECs

To determine whether *M. bovis* infection affects cellular autophagy, transmission electron microscopy (TEM) was used to assess ultrastructure of bMECs infected with the *M. bovis* strain PG45. Numbers of autophagosome-like membrane vesicles were increased in the cytoplasm of *M. bovis*-infected bMECs (Figures 1Aa–c), with some *M. bovis* sequestered in these membrane vesicles (Figure 1Ad). In contrast, similar vesicles were rarely seen in uninfected cells (Figure 1Aa). To further determine whether autophagy can be triggered by *M. bovis* infection, we next examined bMECs autophagy-associated protein LC3, SQSTM1/P62, Beclin1, ATG5 levels, important hallmarks of autophagy, using Western blotting analyses. LC3-II protein levels were increased at 3 h of *M. bovis* infection (Figures 1B–D). Levels of SQSTM1 protein were decreased in infected cells at 3 h (Figures 1B,C,E). Furthermore, Beclin1 was higher at 3 h (Figures 1B,C,F).

In addition, we further determined the localization relationship between the autophagosomal marker LC3B and *M. bovis* in bMECs by confocal laser microscopy. *M. bovis* infection significantly enhanced LC3B (green) punctate staining signals distributed throughout the entire cytoplasm (Figure 1G), whereas control bMECs exhibited a faint diffuse staining pattern and limited LC3B punctate accumulation. Moreover, *M. bovis* (stained red with DiI) signals in infected bMECs displayed punctate accumulation, and the *M. bovis* red fluorescent punctate staining was highly co-localized with LC3B green fluorescent punctate staining. Thus, *M. bovis* infection was capable of causing autophagy in bMECs.

### The role of PTEN/PI3K-Akt-mTOR pathway in *Mycoplasma bovis* inhibiting autophagy in bMECs

The PI3K, Akt and mTOR kinase-dependent signaling pathway controls autophagy. The PTEN protein, a phosphatase for the second messenger phosphatidylinositol 3,4,5-triphosphate, is required for activation of the Akt kinase in the PI3K-Akt pathway. Decreased SQSTM1 protein at 3 h was attributed to autophagic degradation, whereas gradually restored levels of SQSTM1 implied that the autophagy flux was blocked at 6 to 12 h (Figure 1E). Meanwhile, LC3-II and Beclin1 protein expression levels returned to normal at 6 h of infection (Figures 1D,F). To determine whether PTEN/PI3K-Akt-mTOR modulated autophagy upon *M. bovis* infection, the PTEN, PI3K, Akt and mTOR activity in host cells were determined. Compared to control bMECs, PI3K, Akt and mTOR phosphorylation in *M. bovis*-infected bMECs was increased at 6 h (Figures 2A,B,D–F). However, PTEN did not remain increased at 0 to 9 h (Figures 2A–C). Therefore, *M. bovis* inhibited autophagy in bMECs by activating the PI3K-Akt-mTOR signaling pathway.

### Phosphorylation of Akt is positively related to the inhibition of autophagy by *Mycoplasma bovis*

The association between PI3K basal activity and *M. bovis* inhibition of autophagy by modulating PI3K-dependent signaling in bMECs and effects of *M. bovis* infection of bMECs on autophagy were investigated. To decrease PI3K signaling in bMECs without irreversibly inhibiting Akt phosphorylation, before infection, cells were treated with the pan-ErbB inhibitor CI-1033. Based on the CCK-8 assay, CI-1033 did not reduce bMECs viability (Figure 4A). Furthermore, CI-1033 decreased the amount of p-Akt without significantly affecting the total amount of Akt, indicating that CI-1033 specifically inhibited Akt phosphorylation and thereby PI3K signaling in bMECs. In CI-1033 pretreated bMECs, LC3-II protein expression was significantly increased, SQSTM1 protein expression was significantly decreased, and cellular autophagy was activated (Figures 4B,C). When protein expression levels of bMECs pretreated with *M. bovis* infection CI-1033 were measured, p-Akt protein expression was re-elevated and p-Akt was re-activated, whereas LC3-II protein expression levels were significantly decreased and SQSTM1 protein expression levels were significantly increased (Figures 4B,C). The relative mRNA expression levels of Akt and autophagy-related genes were detected by real-time PCR; there was no significant difference between groups in the mRNA expression levels of Akt. In addition, SQSTM1 mRNA levels were significantly lower in the *M. bovis* infection CI-1033 pretreatment group compared to the CI-1033 alone treatment group (Figure 4D). Therefore, after reducing PI3K signaling without completely inhibiting PI3K function, *M. bovis* infection activated phosphorylation of Akt and inhibited autophagy of bMECs.

### Inhibition of Akt with specific siRNAs targeting Akt reverses the inhibition of autophagy in bMECs by *Mycoplasma bovis*

To further verify impacts of Akt on *M. bovis*-inhibit bMECs autophagy, bMECs were transfected for 48 h with small interfering (siRNA)-control (NC) or siRNA-Akt. After transfection with siRNA-Akt-1, siRNA-Akt-2 and siRNA-Akt-3, protein levels of Akt were significantly decreased compared to control and NC-transfected cells, indicating that expression of the Akt proteins was successfully inhibited. Furthermore, as siRNA-Akt-1 had the highest inhibitory efficiency for Akt among the 3 siRNA-Akt groups (Figure 5A), it was used to inhibit Akt expression (designated siRNA-Akt).

Based on Western blotting, in bMECs infected with *M. bovis* after siRNA-Akt transfection, downregulation of Akt by siRNA markedly suppressed *M. bovis*-induced activation of p-Akt. Meanwhile, expression levels of Beclin1 and LC3-II proteins were elevated, and the expression level of SQSTM1 protein was decreased (Figures 5B,C). The relative mRNA expression levels of Akt and autophagy-related genes were detected by real-time PCR; siRNA-Akt transfection down-regulated the mRNA expression level of Akt. Meanwhile, bMECs after *M. bovis* infection with siRNA-Akt knockdown had LC3, Beclin1 and SQSTM1 mRNA levels significantly upregulated compared to the *M. bovis*-infected group. (Figure 5D). Therefore, downregulation of Akt expression with siRNA targeting activated autophagy in bMECs and this activation was not reversed by bovine mycoplasma infection.

### Absence of PTEN expression exacerbates the inhibition of autophagy of bMECs by *Mycoplasma bovis*

To determine effects of activation of the PI3K-Akt-mTOR signaling pathway on autophagy in *M. bovis*-infected bMECs, we knocked down PTEN, a phosphatase that dephosphorylates PIP3 into PIP2, to ensure steady PI3K signaling. The bMECs were transfected for 48 h with small interfering (siRNA)-control (NC), siRNA-PTEN-1, siRNA-PTEN-2 and siRNA-PTEN-3, and PTEN depletion assessed by Western blotting. As siRNA-PTEN-1 had the highest inhibitory efficiency for PTEN (Figure 6A), it was used to inhibit PTEN (designated siRNA-PTEN). Based on Western blotting, siRNA-PTEN strongly increased p-Akt, indicating that PI3K signaling is activated under conditions of PTEN knocked down. After downregulation of PTEN with siRNA, *M. bovis* infection significantly increased *M. bovis*-induced activation of p-Akt. Furthermore, expression of Beclin1 and LC3-II proteins decreased, but expression of SQSTM1 protein also decreased compared to the control group (Figures 6B,C). Based on real-time PCR, loss of PTEN inhibited transcription of autophagy-related genes (e.g., Beclin1, LC3-II) to varying degrees. However, only Beclin1 mRNA levels were down-regulated, whereas LC3 mRNA levels were up-regulated and SQSTM1 mRNA levels were not significantly changed after *M. bovis* infection with bMECs knockdown by siRNA-PTEN compared to the *M. bovis*-infected group (Figure 6D). Therefore, downregulation of PTEN expression exacerbated the inhibition of autophagy in bMECs by bovine mycoplasma infection.

### PTEN promotes autophagy of bMECs by inhibiting PI3K-Akt-mTOR pathway to suppress replication of *Mycoplasma bovis*

To explore effects of PI3K-Akt-mTOR signaling pathway activation on *M. bovis* survival in bMECs, co-localization of LC3 and *M. bovis* were assessed by confocal laser microscopy and intracellular *M. bovis* enumerated. We used siRNA-Akt and siRNA-PTEN to knock down expression of Akt and PTEN in bMECs. After transfection with siRNA-Akt and siRNA-PTEN for 48 h, bMECs were infected with *M. bovis*, there was co-localization of LC3B and *M. bovis* in bMECs; furthermore, co-localization of LC3B and *M. bovis* increased after downregulating Akt expression (Figure 3A). In contrast, co-localization of LC3B and *M. bovis* was decreased after downregulating PTEN expression (Figure 3A). Subsequently, intracellular proliferation of *M. bovis* was detected at 3, 6, 12, and 24 h post-infection. Intracellular *M. bovis* was significantly decreased at 12 and 24 h post-infection in the siRNA-Akt group relative to the *M. bovis* infection group (Figure 3B). However, there were considerably more *M. bovis* in the siRNA-PTEN group at 12 and 24 h post-infection (Figure 3B). Taken together, our results indicated a causal relationship between the Akt and PTEN status of bMECs and their permissiveness to intracellular pathogens.

## Discussion

Autophagy, a common non-selective self-digestion process in cells (Riebisch et al., 2021), is also a highly selective defense against intracellular pathogens (Mostowy, 2013). However, various pathogenic microorganisms have evolved to evade and utilize cellular autophagy (Choy and Roy, 2013; Huang and Brumell, 2014). For example, *M. bovis* inhibited phagosomal-lysosomal maturation to evade cellular autophagy (Liu et al., 2021). In the present study, *M. bovis* activated the early autophagic response in bMECs and inhibited autophagy by activating the PI3K-Akt-mTOR signaling pathway at later stages of infection, whereas downregulation of PTEN gene expression promoted replication of *M. bovis* in bMECs.

We established an *in vitro* autophagy model of *M. bovis* infection with bMECs, based on the ability of *M. bovis* to invade a variety of cells, including lymphocytes and epithelial cells (van der Merwe et al., 2010; Liu et al., 2021). It is noteworthy that *M. bovis* invades cells, evades cellular immunity and the killing effect of some antibiotics, promoting persistent infections and transmission (Bürki et al., 2015). There is accumulating evidence that *Mycoplasma* infection is closely associated with autophagy (Lu et al., 2017; Luo et al., 2020; Liu et al., 2021). In the present study, membrane-like material was wrapped around *M. bovis*, perhaps due to fusion of *M. bovis* with autophagic vesicles, consistent with previous findings of autophagy targeting *M. bovis* (Liu et al., 2021). In addition, upregulated expression of LC3-II, Beclin1 proteins and reduced expression of SQSTM1 protein (LC3-II, Beclin1, and SQSTM1 are the most widely recognized molecular indicators of autophagy) during the early stages of *M. bovis* infection with bMECs. Our findings were consistent with a report that both *Mycoplasma ovipneumoniae* and *Mycoplasma bovis* infections altered expression levels of proteins critical for cellular autophagy (Luo et al., 2020; Liu et al., 2021). *Staphylococcus aureus* infection upregulated LC3-II expression and GFP-LC3B clustered around intracellular bacteria (Geng et al., 2020). In this study, GFP-LC3B was co-localized with *M. bovis* and LC3-II expression was upregulated. Autophagy is an important player in cellular protection against pathogenic infection (Deretic, 2021). Therefore, in the present study, *M. bovis*-invading cells successfully induced autophagy in bMECs at an early stage. However, from 6 h of *M. bovis* infection, LC3- II and Beclin1 protein expression in bMECs was reduced. Thereafter, SQSTM1 protein levels gradually recovered, indicating that degradation of SQSTM1 protein was inhibited. Under normal circumstances, SQSTM1 is a link between LC3 and substrates, with SQSTM1 and SQSTM1-bound substrates incorporated into the completed autophagosome during the initial stage and subsequently degraded in autolysosomes (Hua et al., 2015). Therefore, autophagy was inhibited in mid and late stages of *M. bovis* infection, consistent with inhibition of cellular autophagy by *M. bovis* (Liu et al., 2021).

Despite the key role of autophagy to control pathogens, the latter have also adopted many defense mechanisms to defend against and exploit cellular autophagy (Choy and Roy, 2013; Huang and Brumell, 2014; Li et al., 2015; Sudhakar et al., 2019). However, molecular mechanisms by which *M. bovis* evades cellular autophagy are largely unknown. Phosphatidylinositide 3-kinases (PI3Ks) have key roles in regulation of autophagy. PtdIns (3,4,5) P3, the product of class I PI3Ks, trigger the mTOR signalling pathway, which inhibits autophagy (Farrell et al., 2013). In addition, mTOR, a highly conserved kinase in the phosphatidylinositol 3-kinase family with serine/threonine activity, responds rapidly to various environmental cues and regulates cellular metabolism and immune responses by modulating the kinase Akt (Cobbold, 2013). Activation of the PI3K-Akt-mTOR signaling pathway inhibits the autophagy required for development and survival of nutrient deprivation (Zhao et al., 2019). In the present study, PI3K-Akt–mTOR was activated by phosphorylation in the middle phase of *M. bovis* infection. Invasion of *M. tuberculosis* also initiated the PI3K-Akt-mTOR signaling pathway (Lachmandas et al., 2016). Furthermore, inhibition of autophagy by *M. bovis* in bMECs requires phosphorylation activation of Akt. Treatment of bMECs with pan-ErbB inhibitor CI-1033 inhibited Akt phosphorylation and also inhibited the autophagic activity of bMECs. It was surprising that Akt was activated by rephosphorylation and autophagy was subsequently inhibited when *M. bovis* infected bMECs. Furthermore, following downregulation of Akt expression by small interfering RNA, the autophagic flux of bMECs was increased and cellular autophagy was activated, and this activation of autophagy was not inhibited by *M. bovis* infection. *Streptococcus agalactiae* infection may inhibit PI3K-Akt-mTOR signaling, thereby affecting the autophagic response (Qi et al., 2022). Furthermore, Akt also has an important role in mediating infection of epithelial cells and macrophages by *Salmonella* (Reggio et al., 2020). Several hypotheses have been proposed to explain the mechanism by which Akt promotes bacterial intracellular proliferation or survival, including the idea that by accelerating the recycling of the RAB14 small G protein, Akt delays phagosome-lysosome fusion and thus may enhance bacterial intracellular survival (Kehl et al., 2020). Therefore, we inferred that *M. bovis* inhibited bMECs autophagy and promoted intracellular survival of bMECs through activation of the PI3K-Akt-mTOR signaling pathway.

Activation of PI3K-Akt-mTOR phosphorylation was regulated by multiple upstream proteins, with the tumor suppressor PTEN protein required for activation of Akt/PKB kinase in the PI3K-Akt pathway (Dempsey et al., 2021; He et al., 2021). PTEN is a second messenger phosphatidylinositol 3,4,5-trisphosphate phosphatase that catalyzed conversion of PIP3 to PIP2 through its acidase activity, thereby regulating activity of Akt and mTOR (Chia et al., 2015). Competent PTEN protein is apparently not only critical for tumor suppression but also for resisting infection of mammalian cells by *Mycobacterium bovis* Bacillus Calmette-Guérin (BCG) and Mycoplasma, 2 pathogens with distinct pathogenic strategies (Huang et al., 2012). However, whether the presence of PTEN genes affects the regulation of PI3K-Akt-mTOR by *M. bovis* and thus alters the autophagic state of bMECs, is unclear. In the present study, small interfering RNAs downregulated PTEN expression and Akt phosphorylation was maintained, which in turn inhibited autophagy of bMECs, whereas the co-localization between GFP-LC3B and *M. bovis* was reduced, consistent with the findings of Wang et al. (2021a). However, the decreased SQSTM1 protein may be attributed to inhibition of SQSTM1 protein production by Akt phosphorylation. To confirm effects of downregulation of PTEN and inhibition of PI3K-Akt-mTOR signaling pathway on intracellular *M. bovis*, bMECs were treated with small interfering RNA PTEN or small interfering RNA Akt; the intracellular number of *M. bovis* was related to the level of autophagy in the host cells induced by downregulation of PTEN or disruption of Akt signaling was inversely related to changes in the level of autophagy in host cells caused by downregulation of PTEN or disruption of Akt signaling, consistent with previous reports for *Mycoplasma bovis* (Liu et al., 2021). Furthermore, downregulation of the PTEN gene increased intracellular *M. bovis* replication, consistent with previous reports that downregulation of PTEN gene exacerbated *Mycobacterium bovis* Bacillus Calmette-Guérin (BCG) and *Mycoplasma* infection and proliferation (Huang et al., 2012). Therefore, we inferred that PTEN promoted autophagy during *M. bovis* infection of bMECs by inhibiting the PI3K-Akt-mTOR pathway to suppress survival and replication of *M. bovis*.

Although important discoveries were revealed by these studies, there are still limitations. In this study, only *in vitro* models were used to assess effects of intracellular *M. bovis* on autophagy and autophagic signaling pathways. However, it is well known that various cellular interactions *in vivo* give rise to more complex mechanisms.

## Conclusion

**Figure 7:**
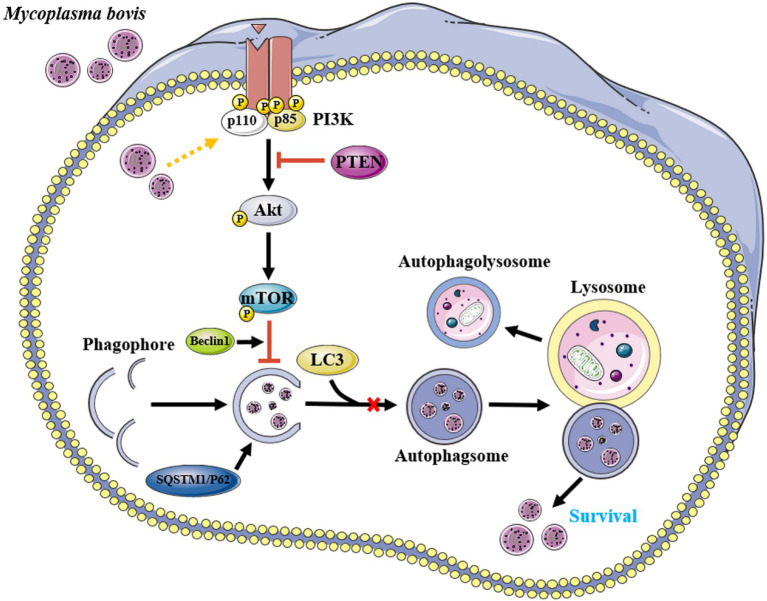
Proposed model for inhibition of autophagy by *M. bovis* via the PTEN/PI3K-Akt-mTOR pathway. Infection of invading bMECs by *M. bovis* activated phosphorylation of Akt and inhibited the onset of downstream autophagic activity, preventing autophagosomes from delivering engulfed *M. bovis* to lysosomes for degradation, thereby enabling *M. bovis* to survive. Yellow arrow and red bar indicate stimulation and inhibition, respectively. This figure was partially made using Servier Medical Art (smart.servier.com).

*M. bovis* infection activated the PI3K-Akt-mTOR signaling pathway, inhibited autophagy of bMECs, and promoted their survival and replication, whereas PTEN has an important role in cellular autophagy caused by *M. bovis* infection (Figure 7). These findings provided new insights into molecular mechanisms enabling *M. bovis* to evade cellular autophagy. Perhaps targeted regulators of autophagy will be developed to better understand intracellular pathogens, with potential to increase vaccine efficacy.

## Data availability statement

The original contributions presented in the study are included in the article/supplementary material, further inquiries can be directed to the corresponding author.

## Author contributions

BH and MX conceived and designed the experiment. MX, YLu, TM, YLn, YW, JG, and DW performed the research and wrote the manuscript. JK and TM assisted in the analyses and re-edited the manuscript. JK and BH revised the manuscript. All authors contributed to the article and approved the submitted version.

## Funding

This study was supported financially by the National Natural Science Foundation of China (Nos. 32172928 and 31760751), Ningxia Key R&D Project (No. 2019BBF02027), the High-End Foreign Experts Recruitment Program (No. GDT20171100013), and the Natural Science Foundation of Ningxia (No. 2022AAC02022).

## Conflict of interest

The authors declare that the research was conducted in the absence of any commercial or financial relationships that could be construed as a potential conflict of interest.

## Publisher’s note

All claims expressed in this article are solely those of the authors and do not necessarily represent those of their affiliated organizations, or those of the publisher, the editors and the reviewers. Any product that may be evaluated in this article, or claim that may be made by its manufacturer, is not guaranteed or endorsed by the publisher.
